# Energetic Performance of Pure Silica Zeolites under High-Pressure Intrusion of LiCl Aqueous Solutions: An Overview

**DOI:** 10.3390/molecules25092145

**Published:** 2020-05-04

**Authors:** Giorgia Confalonieri, T. Jean Daou, Habiba Nouali, Rossella Arletti, Andrey Ryzhikov

**Affiliations:** 1 Axe Matériaux à Porositées Contrôlées, Université de Haute Alsace (UHA), CNRS, IS2M UMR 7361, F-68100 Mulhouse, France; giorgia.confalonieri@unimore.it (G.C.); habiba.nouali@uha.fr (H.N.); 2 Université de Strasbourg, F-67081 Strasbourg, France; 3 Dipartimento di Scienze Chimiche e Geologiche (DSCG), Università di Modena e Reggio Emilia, 41125 Modena, Italy; rossella.arletti@unimore.it

**Keywords:** pure silica zeolites, zeosils, high-pressure intrusion, electrolyte aqueous solutions, mechanical energy absorption and storage, heterogeneous lyophobic systems

## Abstract

An overview of all the studies on high-pressure intrusion—extrusion of LiCl aqueous solutions in hydrophobic pure silica zeolites (zeosils) for absorption and storage of mechanical energy is presented. Operational principles of heterogeneous lyophobic systems and their possible applications in the domains of mechanical energy storage, absorption, and generation are described. The intrusion of LiCl aqueous solutions instead of water allows to considerably increase energetic performance of zeosil-based systems by a strong rise of intrusion pressure. The intrusion pressure increases with the salt concentration and depends considerably on zeosil framework. In the case of channel-type zeosils, it rises with the decrease of pore opening diameter, whereas for cage-type ones, no clear trend is observed. A relative increase of intrusion pressure in comparison with water is particularly strong for the zeosils with narrow pore openings. The use of highly concentrated LiCl aqueous solutions instead of water can lead to a change of system behavior. This effect seems to be related to a lower formation of silanol defects under intrusion of solvated ions and a weaker interaction of the ions with silanol groups of zeosil framework. The influence of zeosil nanostructure on LiCl aqueous solutions intrusion–extrusion is also discussed.

## 1. Introduction

### 1.1. Heterogeneous Lyophobic Systems

Nowadays, an efficient energy transformation and storage is one of the main technological challenges of the world. Heterogeneous lyophobic systems (HLSs), i.e., systems composed by a nanoporous solid and a nonwetting liquid, have attracted much attention as promising candidates for innovative mechanical energy storage and dissipation devices [1,2]. In these systems, mechanical energy (i.e., an external pressure) is required to force the intrusion of a non-wetting liquid into the pores of material. Indeed, the penetration of the liquid occurs only when the applied external pressure is higher than the capillary pressure of the porous matrix, defined as Equation (1) by the Laplace–Washburn relation [3]:

(1) $$P_{c}=-4\gamma_{L}cos\theta /D$$

where *P_c_* is the capillary pressure, *γ_L_* the liquid-gas surface tension, *D* the diameter of the pore, and *θ* the contact angle between solid surface and liquid (*θ* > 90°).

The penetration of the liquid inside the pores strongly increases the liquid–solid interface area leading to a conversion of mechanical energy, supplied by pressure, into the breaking of intermolecular bonds of the liquid and the interactions at the liquid–solid interface. In the case of microporous materials, during the intrusion, the bulk liquid is transformed to molecular chains and clusters inside the pores. Such a process can be described as capillary evaporation. The absorbed energy during the intrusion process can be expressed as a work and described as Equation (2):

(2) $$W=\int_{V_{0}}^{V_{f}}-P\;dV$$

where *P* is the applied pressure, and *V_0_* and *V_f_* are respectively the initial and the final volume of the system.

**Figure 1:**
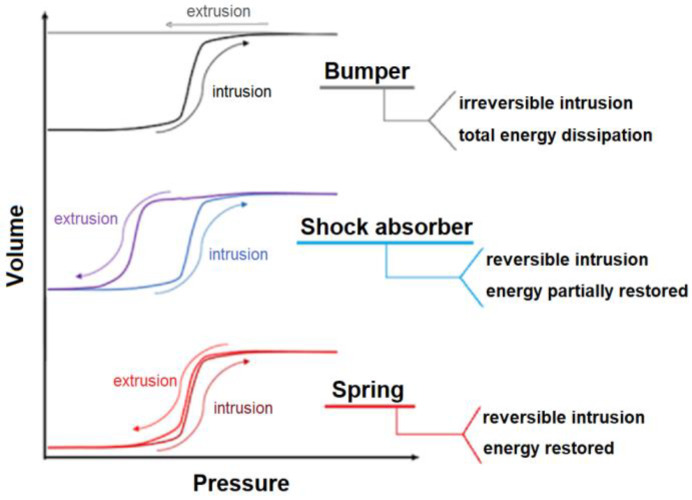
Schematic representation of three main behaviors of heterogeneous lyophobic systems.

When the external pressure is released, the liquid can be extruded, completely or partially, or can remain trapped in the solid. Therefore, the whole or a part of the initial energy is restored, or it is entirely absorbed. Consequently, the system can display a spring or shock-absorber or bumper behaviour or a combination of them. A schematic example of different behaviors of HLSs is shown in Figure 1.

### 1.2. Potential Applications of Heterogeneous Lyophobic Systems

Heterogeneous lyophobic systems with shock-absorber and bumper behavior can be used or integrated in devices aiming to dissipate mechanical energy, for example, for new types of dampers for the automotive and aerospace industries [2,4,5,6,7,8]. Their use as shock absorbers in cars and trucks is very promising, since their energy absorption efficiency is much higher than the one of common hydraulic dampers. Moreover, the shock absorbers based on lyophobic systems should provide a very high comfort level because of their excellent damping coefficient and very long lifetime [4,7]. The HLS can also be exploited for other energy dissipation applications in bumpers, anti-seismic, anti-vibration, and blast protections [2,8,9,10,11,12,13,14].

The heterogeneous lyophobic systems with spring behavior are promising for applications in mechanical energy storage. For example, they could be used in the field of transport (kinetic energy recuperation), in the sources of renewable energy, and as an alternative to common springs, for example, as self-contained actuators for space applications [8].

Due to the rapid decrease of intrusion pressure with temperature increase [15], the systems (porous solid–non-wetting liquid) with spring behavior can also be used for the generation of mechanical energy from low potential (“waste”) heat. In this case, the system works as a heat engine (so-called thermomolecular engine) with a specific thermodynamic cycle and becomes a source of renewable energy [16,17,18,19]. HLS can also be used for other particular applications, such as volume-memory materials [20] or as materials with extremely high negative expansion coefficient [21].

### 1.3. Heterogeneous Lyophobic Systems Based on Hydrophobic Zeolites

The first systems developed by V. Eroshenko in the mid-1980s were based on porous silica and mercury or liquid metallic alloys as non-wetting liquids [22]. Later, water was found to be a more suitable liquid for HLSs because of its nontoxicity, low cost as well as quite high liquid–vapor surface tension and a small kinetic diameter of 2.8 Å which allows the penetration into tiny micropores. However, such HLSs require highly hydrophobic porous materials. The first experiments were performed on mesoporous silica grafted with alkyl and perfluoroalkyl chains [23,24,25]. In 2001, the first use of pure-silica zeolites as hydrophobic solids for mechanical energy storage was reported [26]. Zeolites are microporous crystalline solids with a framework composed by TO_4_ (T = Si, Al, Ge…) tetrahedral units that form channels or cavities. At the moment, 248 different zeolitic frameworks are known. Each of them is identified by a three letter code assigned by the International Zeolite Association. These materials are widely used in adsorption, catalysis, molecular sieving, and ion exchange [27]. Pure silica zeolites (zeosils), particularly the ones obtained in fluoride medium, are known to have highly hydrophobic character and thus are of high interest for the use in heterogeneous lyophobic systems. Many zeosils pertaining to different framework types were studied in high-pressure water intrusion–extrusion experiments [28,29,30,31,32,33,34,35,36,37,38,39,40]. Due to the sub-nanometer pore diameter of these materials, extremely high values of water intrusion pressure, up to 210 MPa, and, consequently, of stored energy, up to 15 J/g [28,29], are achieved. It was observed that energetic performance depended strongly on zeosil structure. Moreover, the intrusion–extrusion characteristics and, particularly, the behavior of the system, are influenced by the presence of silanol defects or their formation under water intrusion.

According to Equation (2), the energetic performance of HLSs can be improved by an increase of intrusion pressure. One of the promising ways to increase the intrusion pressure is the use of electrolyte aqueous solutions as non-wetting liquid instead of water. The pressure rise with the increase of salt concentration was observed for different salt solutions [41,42,43]. This effect is particularly pronounced for highly concentrated solutions, where the number of water molecules becomes close or lower than the coordination number of salt cations and anions [44]. In such solutions, the nature of anions and cations has a considerable influence on intrusion–extrusion characteristics, whereas its influence is much lower for the diluted solutions [45]. For instance, the highest increase of intrusion pressure, by more than seven times, was observed for the intrusion of saturated LiCl aqueous solution in LTA-type zeosil [46]. The case of LiCl electrolyte aqueous solutions is particularly interesting because of very high solubility of this salt that makes it possible to achieve a very high molar concentration (up to 20 M for the saturated aqueous solution) with a very low H_2_O/salt molar ratio (2.8). Due to these reasons and to a particularly strong effect on intrusion pressure, the intrusion of LiCl aqueous solutions was studied for different zeosils with various framework types [29,44,46,47,48,49,50,51,52,53,54,55], whereas the aqueous solutions of other salts are quite poorly studied at the moment. In this paper, we focus only on the intrusion—extrusion of LiCl aqueous solutions in zeosils and present an overview of all the results reported in order to discuss the main relationships between zeolite structure and energetic performance of corresponding HLS.

## 2. Water Intrusion in Zeosils

In order to introduce an overview of high-pressure intrusion of LiCl aqueous solutions, we describe briefly the main results of intrusion–extrusion experiments for “zeosil–water” systems. For all the frameworks considered, the average pore diameter values calculated from corresponding CIF (Crystallographic Information File) files and maximal diameter of the sphere which can be included in the microporosity are presented in Table 1 [56]. The studies of water intrusion—extrusion were performed for many zeosils with different framework types [26,28,29,30,31,32,33,34,35,36,37,38,39,40]. The results obtained for some of these zeosils (lines for the concentration of 0 M) are summarized in Table 2 along with the results obtained for intrusion of LiCl aqueous solutions. In this table, the zeosils are classified by type of porous system (cages or channels) and its dimensionality (1D, 2D, 3D). This latter parameter is of great importance. In fact, it has been observed that for the systems based on channel-type zeosils, the intrusion pressure depends on the channel diameter, whereas for the cage-type ones, it does not correlate with the diameter of pore openings but is related to the cage size (i.e., its maximal diameter) or the size of the includible sphere [28,57,58]. For the cage-type zeosils, the water fills the porosity at relatively low pressure values (20–60 MPa). Conversely, for the channel-type ones, the intrusion pressure is generally higher—up to 210 MPa [28,29]. Overall, the intrusion pressure increases with the decrease of channel/cage diameter.

The highest water intrusion pressures have been obtained for 1D and 2D channel-type zeosils with relatively small channel diameter. The maximal value (210 MPa) was observed for CDO-type zeosil (two-dimensional channels, eight member-ring (MR) pore openings) [29]. For TON- and MTT-type zeosils (1D channels, 10 MR), the intrusion pressure reaches 180 and 176 MPa, respectively [30,32]. Nevertheless, since the absorbed/stored energy depends not only on intrusion pressure, but also on intruded volume, the highest value of absorbed energy (15 J/g) is obtained for AFI-type zeosil which couples a quite high intrusion pressure (132 MPa) and a high intruded volume (0.12 mL/g) [30]. It should be noticed that TON-, MTT-, and AFI-type zeosils have not been studied yet in intrusion—extrusion experiments with LiCl solutions.

The presence of hydrophilic silanol defects (i.e., Si-OH) in zeosils leads to a lower value of intrusion pressure. A difference of intrusion–extrusion pressure of MFI-type zeosil (silicalite-1) prepared in F^−^ and OH^−^ medium was demonstrated in the works of Eroshenko et al. and Trzpit et al. [26,59]. In fact, the synthesis in OH^−^ medium, leding to higher silanol content, showed a decrease of intrusion pressure with respect to that synthesized in F^−^ (from 99 to 81 MPa) [26]. Another example is observed for CFI- and DON-type zeosils. These zeosils have a close channel diameter (1D channels, 14 MR for both), but show a strong difference of intruded pressure values (75 and 26 MPa, respectively), since the DON-type zeosil has a higher content of silanol groups [50]. It is worth noting that if a high number of hydrophilic sites is present in the cavities, water fills the pores spontaneously and no mechanical energy is absorbed, as it occurs in OKO-type zeosil. The presence of hydrophilic defects can also impact the intrusion reversibility and, thus, intrusion–extrusion behavior. This aspect is discussed below in Section 4.

## 3. Influence of LiCl Aqueous Solutions on Intrusion Pressure

**Figure 2:**
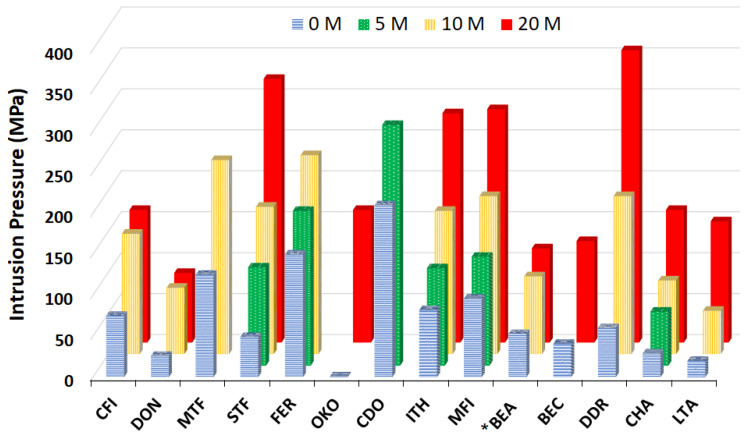
Intrusion pressure values of different “zeosil–LiCl aqueous solution” systems in function of solution concentration of 0 (water), 5, 10, and 20 M.

**Figure 3:**
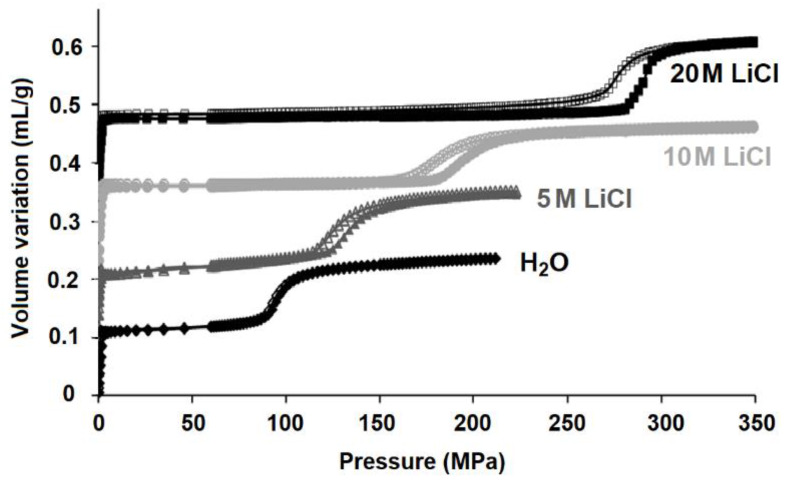
Intrusion–extrusion curves of “MFI-type zeosil–H_2_O” and “MFI-type zeosil–LiCl aqueous solution” systems. The results are taken from [47].

Intrusion–extrusion characteristics of different zeosils intruded by LiCl aqueous solution at different concentrations (0, 5, 10, and 20 M) are summarized in Table 2. A graphical comparison of their intrusion pressure values is given in Figure 2. It can be observed that the values vary considerably as a function of zeosil framework. Overall, independently from the type of pore system and pore size, the intrusion pressure increases with increasing LiCl concentration. An example of the evolution of intrusion–extrusion curves with the concentration is presented in Figure 3 for MFI-type zeosil. The highest intrusion pressure is observed for “DDR-type zeosil—20 M LiCl aqueous solution” system (357 MPa). High values were also obtained for the intrusion of 20 M LiCl aqueous solution in STF-, MFI-, and ITH-type zeosils at 322, 285, and 280 MPa, respectively. No high pressure intrusion step was found in the case of 20 M LiCl aqueous solution and FER-, MTF-, and CDO-type zeosils, whereas it is well observed for the solutions with a lower concentration of LiCl. This phenomenon can be explained by the limit of 400 MPa in the pressure that can be applied by the used device. Thus, it can be reasonably supposed that for these materials, the intrusion pressure should be superior to 400 MPa.

On the basis of the data reported, the following hypotheses are proposed to explain the observed increase of the intrusion pressure: (i) the rise of surface tension of aqueous electrolyte solution in comparison with water according to Laplace–Washburn Equation (1); (ii) osmotic phenomena [60]; (iii) the confinement effect of nanopore walls [61]; and (iv) the desolvation of solvated ions and the deformation of their solvation sphere during the penetration into the pores [46]. The first hypothesis alone cannot explain a strong rise of intrusion pressure in zeosils, since the increase of surface tension is about 35% from water (72.8 mN/m) to 20 M LiCl aqueous solution (98 mN/m). Indeed, according the studied systems, the pressure increase is more or less marked, but in any case, it is equal for all the zeosils [62]. The second and the third ones seem to be valid only for diluted solutions. Thus, the desolvation and the distortion of hydrated ions should mainly be responsible for the pressure increase. The ions solvated by water molecules can penetrate inside sub-nanometer pores of zeosils only after a partial desolvation and a deformation of their solvation sphere. Therefore, more energy is required for this process in comparison with the intrusion of water. The penetration of solvated ions into the pores after partial desolvation was demonstrated by in situ high pressure X-ray powder diffraction (HP XRPD) studies on several “zeosil–salt aqueous solution” systems. The first structural study on FER-type zeosil and MgCl_2_•21H_2_O solution demonstrated that the intruded liquid did not have the composition of the initial solution but was more concentrated (MgCl_2_•10H_2_O), close to the maximal salt solubility [63]. The intrusion—extrusion process of NaCl, NaBr, and CaCl_2_ aqueous solutions (2M and 3M) was also studied in CHA- and LTA-type zeosils with the same technique and similar results were obtained [63,64,65]. The concentrations of the intruded solutions were considerably higher with respect to the initial ones, confirming the ion desolvation process as the key point of the intrusion of salt aqueous solution in the zeosils. It can be supposed that a similar phenomenon occurs in the case of LiCl aqueous solutions. Unfortunately, because of the low electron density of lithium ion and, thus, its weak atomic scattering power, in situ HP XRPD experiments cannot be performed for “zeosil–LiCl solution” systems.

**Figure 4:**
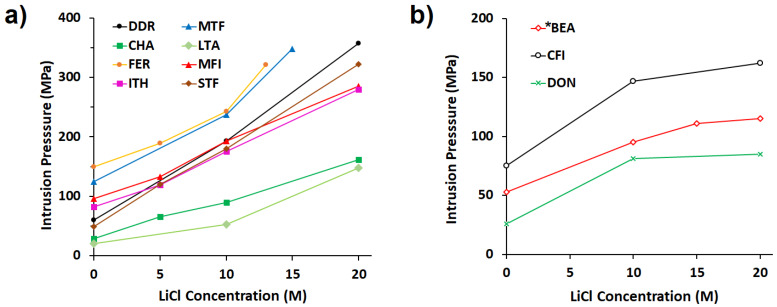
Evolution of the intrusion pressure values with LiCl concentration for (**a**) zeosils with narrow pore openings (8, 9, and 10 MR), (**b**) zeosils with large pore openings (12 and 14 MR). Only the intrusion of samples investigated with at least three different concentrations are considered.

The increase of intrusion pressure with LiCl concentration is not the same for all the zeosils studied as it is shown in Figure 4. It seems that the zeosils with narrow pore openings (8, 9, 10 MR (Figure 4a)) underwent a stronger enhancement of the intrusion pressure with respect to those with larger pores (*BEA, CFI, DON, 12 or 14 MR (Figure 4b)). Moreover, the evolution as a function of the LiCl concentration has a linear or a parabolic trend for narrow pore openings, whereas for the zeosils with large pores after a first linear increase (from 0 to 10 or 15 M), a plateau is almost reached. It could be supposed that under penetration in large pores, the ions should be less desolvated and their hydration sphere less distorted.

**Figure 5:**
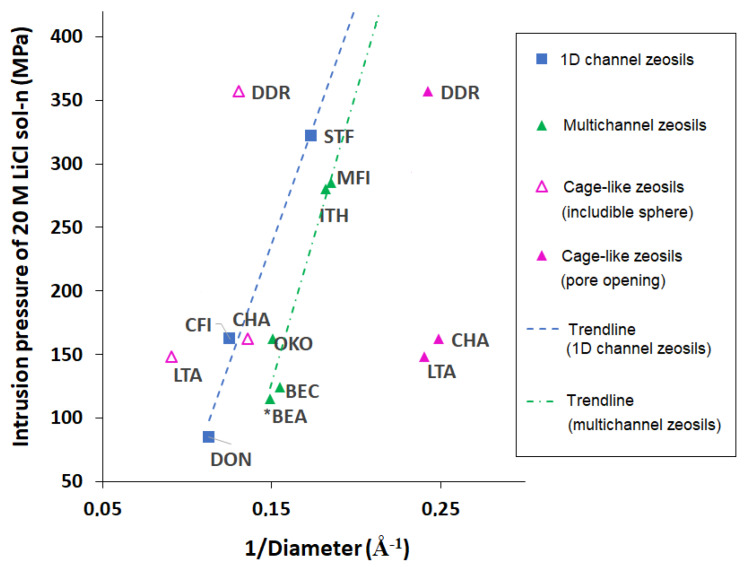
Intrusion pressure of 20 M LiCl aqueous solution versus the inverse of the average diameter of the pores: for 1D and multichannel zeolites as the average of the diameter opening; for cage like zeolites as the maximum diameter of the sphere that can be included in the pores (empty symbol), and as the average dimeter of the pore opening.

It can be concluded that the intrusion pressure of LiCl aqueous solutions depends considerably on the zeosil framework, particularly on the pore size. The evolution of intrusion pressure of saturated LiCl aqueous solution (20 M) as a function of the inverse of pore size (average diameter of pore opening for channel-type zeosils, average diameter of pore opening, and maximal diameter of sphere that can be included in pores for cage-type ones) is presented in Figure 5. As it was mentioned above, in the case of water intrusion, the intrusion pressure is proportional to the inverse of the average pore diameter for channel-type zeosils and of the cage diameter for the cage-type ones. In the case of LiCl aqueous solutions, the situation is not always the same. Firstly, it was difficult to find a correlation between all the zeosils, but some trends can be distinguished when the zeosils of the same type of porosity are considered: 1D channel systems, multichannel (2D and 3D) pore systems, and the cage-type ones. For the channel-type zeosils, the intrusion pressure of 20 M LiCl aqueous solution increases with the decrease of pore opening diameter according to Laplace–Washburn Equation (1) as well as in the case of water. On the contrary, no clear dependence was found, neither for pore opening size nor for the included sphere diameter, for the cage-type zeosils. In spite of a similar size of pore openings, DDR-type zeosil demonstrates a considerably higher intrusion pressure than the LTA- and CHA-type ones. Moreover, no correlation with the maximal diameter of included sphere is observed. LTA- and CHA-type zeosils show similar values of intrusion pressure (148 and 162 MPa, respectively) despite different includible sphere diameter, the DDR-type one demonstrates a much higher intrusion pressure (357 MPa) having a cage size similar to that of the chabazite (CHA).

**Figure 6:**
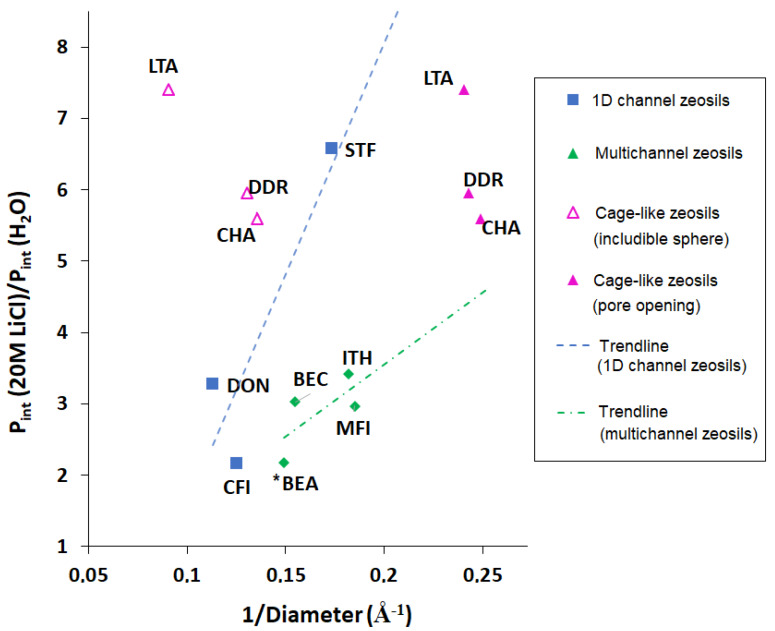
The relative increase of intrusion pressure of 20 M LiCl aqueous solution in comparison with water (P_int_ (20M LiCl)/P_int_ (H_2_O)) versus the inverse of the average diameter of the pore openings for channel-type zeosils and the diameter of pore openings and the maximal diameter of included sphere for cage-type ones.

As well as the absolute intrusion pressure of 20 M LiCl aqueous solution, it is also interesting to compare these values with those obtained with water. The values of relative increase (P_int_ (20 M LiCl)/P_int_ (H_2_O)) for different zeosils are presented in Figure 6. In general, three different tendencies can be observed, respectively, for the zeosils with 1D channels, multidimensional channels, and cage pore systems. The cage-type zeosils show a very high relative increase of intrusion pressure: 7.4 for LTA-, 5.95 for DDR-, and 5.6 for CHA-type, indicating that the relative increase is particularly high for the zeosils with small pore openings (8 MR) and that the size of maximum diameter includible sphere has no impact on the rise of the intrusion pressure

Generally, for the zeosils with larger pore openings (10, 12, and 14 MR), the relative increase is quite low (2.0–3.4) except for the STF-type one (1D channels with side pockets, 10 MR). For this material, the increase by 6.3 times can be related to its unidimensional structure.

It should be noticed that the increase of intrusion pressure is determined not only by a pore diameter, but also by higher non-wetting properties of LiCl aqueous solutions which interact less with the silanol defects of the framework with respect to water. Thus, the zeosils with a higher content of silanol groups can demonstrate a higher relative increase of intrusion pressure. This seems to be the case of DON-type zeosil (1D channels, 14 MR), which shows a higher increase (3.3) in comparison with the similar CFI-type one (2.2), having an even slightly lower pore diameter [50]. In comparison with the latter, DON-type zeosil has a higher content of silanol defects, and thus, it demonstrates a considerably lower water intrusion pressure (26 and 75 MPa, respectively). Probably, this effect takes place also when BEC- and *BEA-type zeosils are compared (the relative increase of 3.0 and 2.2, respectively), where the first one contains a higher number of silanol groups. A more detailed discussion of the role of defects is given in the next section.

## 4. Influence of LiCl Aqueous Solutions on Intrusion–Extrusion Behavior

As it was mentioned above, most of the “zeosil–water” systems demonstrate a fully reversible spring behavior. Nevertheless, some of them show a fully or a partially irreversible intrusion, that corresponds to a bumper behavior or to a combination of the bumper and shock-absorber ones, respectively. The irreversible intrusion is related to a presence of hydrophilic silanol groups in the zeosil framework or to their formation, when the intruded water molecules damage the framework breaking siloxane bridges. ITH- [52], *BEA- [26], BEC- [51], IFR- [33], and LTA-type [46] zeosils demonstrate a fully irreversible bumper behavior under water intrusion, whereas the intrusion is only partially irreversible in the case of CHA- [49] and STF-type [32] zeosils. In the latter cases, a part of the water remains inside the pores adsorbed on hydrophilic silanol groups after the first intrusion, but another part is extruded and can be intruded reversibly in the following cycles.

**Figure 7:**
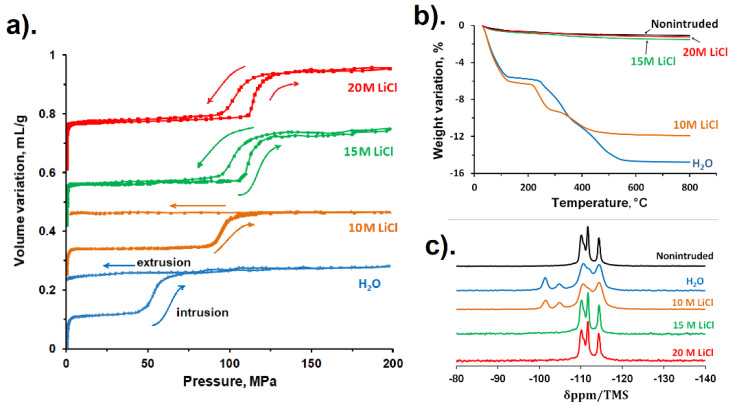
(**a**) Intrusion–extrusion curves for “*BEA-type zeosil–H_2_O” and “*BEA-type zeosil–LiCl aqueous solution” systems. (**b**) Thermogravimetric curves of *BEA-type zeosil samples before and after intrusion–extrusion of water and LiCl aqueous solutions. (**c**) ^29^Si MAS NMR spectra of *BEA-type zeosil samples before and after intrusion–extrusion experiments with water and LiCl aqueous solutions. The results are taken from [44].

Generally, the use of highly concentrated LiCl aqueous solutions instead of water leads to a change of system behavior; specifically, the intrusion becomes more reversible with the rise of LiCl concentration. For the first time, this effect was observed for *BEA-type zeosil [44]. The intrusion of water and LiCl aqueous solutions up to 10 M is fully irreversible (bumper behavior) as it is presented in Figure 7a. Starting from the concentration of 15 M, the intrusion becomes fully reversible, thus, the system demonstrates a spring behavior. This effect is explained by thermogravimetric (TG) analysis and ^29^Si solid-state NMR MAS spectroscopy, which evidenced that silanol groups are not formed under intrusion of highly concentrated solutions. Figure 7b shows the TG curves obtained after drying the powder after the porosimetric experiments. They clearly indicate that in the case of 15 and 20 M LiCl aqueous solutions, the TG weight loss is very low and close to that observed for a non-intruded sample, whereas it is much stronger for the samples intruded with water and 10 M LiCl aqueous solution. The results of NMR spectroscopy confirm this conclusion: the resonances at −100 and −103.5 ppm corresponding to Q_3_ sites ((SiO_3_)SiOH or (SiO_3_)SiO^−^ groups) are clearly observed for the samples after water and 10 M LiCl aqueous solution intrusion, but they are absent on the spectra of the samples intruded with highly concentrated solutions (Figure 7c). The same effect was observed for LTA-type zeosil: a bumper behavior in the case of water intrusion becomes partially reversible (combination of bumper and shock-absorber behavior) in the first cycle and fully reversible (shock-absorber behavior) in the following ones for 10 and 20 M LiCl aqueous solutions [46]. In the case of BEC-, CHA-, and ITH-type zeosils, the intrusion of concentrated LiCl aqueous solutions leads to the formation of a lower number of defects, but the effect is less pronounced [49,51,52]. Nevertheless, the increase of intrusion reversibility is still observed: from bumper to shock-absorber behavior for BEC-type zeosil, from bumper to a combination of bumper and shock-absorber behavior for the ITH-type one and from a combination of bumper and shock-absorber to spring behavior for pure silica chabazite. The formation of a lower amount of silanol groups under intrusion of highly concentrated LiCl aqueous solutions seems to be related to lower reactivity of intruded species towards zeosil framework. As it was discussed above, in the case of salt aqueous solutions, the intruded liquid is not more water, but solutions with low H_2_O/salt molar ratio. In such solutions, most of the water molecules are included in solvation shells of lithium and chloride ions; thus, they become less reactive in the breaking of siloxane bridges of zeosil framework.

However, in some cases, this explanation is not sufficient. For example, in the case of CHA-type zeosil, as well as for the ITH type, silanol groups are already present in the initial samples, and the difference between silanol content after water and LiCl aqueous solutions intrusion is quite low. Nevertheless, the intrusion reversibility increases for these materials. It can be supposed that the intruded concentrated solutions interact less with the hydrophilic defects and become “less wetting liquid”. Water molecules are already bounded with the ions; thus, they are not adsorbed on silanol groups and expelled from the pores under pressure release.

This effect is particularly strong for OKO-type zeosil [55]. Due to a considerably high number of silanol defects, this zeosil is quite hydrophilic, the water intrudes spontaneously at ambient pressure. However, high-pressure intrusion–extrusion steps are observed when using 20 M LiCl aqueous solution [55]. The same effect is also observed in the case of aluminosilica FAU- and *BEA-type zeolites with high Si/Al ratio [66]: the intrusion of water is spontaneous, whereas reversible intrusion–extrusion of the LiCl aqueous solutions is observed at high pressure with shock-absorber and spring behavior of corresponding systems.

Another example is a strong increase of intruded volume with LiCl concentration in DON-type zeosil [50]. This zeosil contains a significant amount of silanol defects and demonstrates a quite low volume of intruded water (0.04 mL/g) compared to its micropore volume. It can be supposed that a part of the pores is filled spontaneously by water and only the filling of the hydrophobic part of the pores at high pressure is observed. When the LiCl aqueous solutions are used, the intruded volume strongly increases—up to 0.08 mL/g for 20 M LiCl aqueous solution. This phenomenon is also explained by lower interactions of the ions with the framework; the LiCl solutions become non-wetting liquids for this hydrophilic part of porosity, and the filling of the total pore volume is observed at high pressure.

A slight increase in the volume of intruded LiCl aqueous solutions (+10%–20%) in comparison with water is observed for all the zeosils studied. It should be noticed that the intruded water volume is generally about 60% of the total micropore volume [57]. It can be supposed that this volume increase for the salt solutions is due to a denser organization of solvated ions inside the pores or to a higher capacity of the ions to better fill the pore volume.

In the cases of STF-type (1D channels with side pockets) [54] and DDR-type (2D cages) [53] zeosils, the intruded volume increase is much more pronounced (by 2.3–3.2 times) in spite of the hydrophobic character of both materials. The intruded volume becomes close to the total available micropore volume of the zeosils. The nature of such volume increase remains unclear at the moment. It should be noticed that these two zeosils demonstrate other particularities under intrusion of LiCl aqueous solutions. In the both cases, a shock-absorber behavior with a very large hysteresis between intrusion and extrusion curves (energy yield of ~50%) is observed in the case of 20 M LiCl aqueous solution. A similar effect is found in the case of MTF-type zeosil, where a two-step extrusion with a large hysteresis is observed for 15 M LiCl aqueous solution, whereas in the case of water and 10 M LiCl solution, the zeosil demonstrates a spring behavior [29]. It should be noticed that three above-mentionned zeosils with unusual behavior (strong increase of intruded volume, large hysteresis…) have a cage or a cage-like (channels with side pockets) pore structure.

A slight increase of the intrusion–extrusion hysteresis with increasing LiCl concentration is observed for most of the zeosils that can be seen through the decreasing energy yield values (see Table 2). The nature of this phenomenon remains unclear, but it could be supposed that it is related to the interactions of solvated ions with zeosil framework.

## 5. Influence of Particle Size and Morphology on Intrusion of LiCl Aqueous Solutions

Some studies on the influence of size and shape of zeosil nanostructures on high-pressure intrusion–extrusion characteristics were performed [67,68]. The intrusion–extrusion of water and 20 M LiCl aqueous solution was realized in the nanosheets (2 nm in thickness and 20 nm in length), the nanocrystals (70 nm) and the hierarchically organized honeycomb-like structures (45–50 nm in thickness and 1–2 μm in length) of MFI-type zeosil (silicalite-1) [67]. The main parameter influencing the intrusion process in these materials seems to be a content of silanol groups which varies considerably from one nanostructure to another. Silicalite-1 nanosheets had a hydrophilic character; thus, a spontaneous intrusion of water and LiCl aqueous solution is observed. In the case of nanocrystals and honeycomb-like structures with lower defect content, the intrusion–extrusion characteristics were very close to the ones of micrometric conventional MFI crystals (15–25 µm). Only a slight decrease of intrusion pressure of 20 M LiCl aqueous solution—from 285 MPa (microcrystals) to 281 and 280 MPa for the nanocrystals and the honeycomb-like structures, respectively—was observed.

The role of nanostructure on LiCl aqueous solutions intrusion was more pronounced in the case of silicalite-1 hollow nanoboxes obtained by dissolution–recrystallization of nanocrystals [68]. Such nanoboxes possess large cavities with a size of 100–250 nm as well as regular walls of 15–20 nm of thickness. They were studied in order to improve a stored energy by the increase of intruded volume due to the presence of the mesoporous cavities. In the case of water intrusion, the cavities were filled spontaneously, whereas the micropores of zeosil walls were filled at the pressure similar to the one of microcrystals. However, a drastic effect of the cavities on the behavior and intrusion–extrusion characteristics was observed in the case of 20 M LiCl aqueous solution. In contrast to the microcrystals, the intrusion of LiCl aqueous solution in silicalite-1 nanoboxes is only partially reversible, and the intruded volume increases in the first intrusion–extrusion cycle (0.11 vs. 0.15 mL/g, respectively). The intrusion phase occurs in two steps. The first one (0.07 mL/g), with an intrusion pressure of 98 MPa, is irreversible and corresponds to the filling of mesoporous cavities through the small cracks in the walls. The second intrusion step (0.08 mL/g) corresponds to the reversible intrusion in the micropores of zeosil walls at the intrusion pressure slightly lower than in the microcrystals (273 against 285 MPa). Unfortunately, the stored energy was not improved in this way because of relatively low pressure values and the intrusion irreversibility in the cavities of nanoboxes.

## 6. Energetic Performance of “Zeosil–LiCl Aqueous Solution” Systems

One of the most promising applications of heterogeneous lyophobic systems is a storage of mechanical energy. Due to a strong rise of intrusion pressure and a smaller increase of intruded volume, the use of highly concentrated LiCl aqueous solutions allows to considerably improve the energetic performance of zeosil-based systems. However, the energy storage applications require a spring behavior of “zeosil–non-wetting liquid” systems with small hysteresis between intrusion and extrusion curves, when the absorbed mechanical energy is almost completely restored. Thus, the ability of LiCl aqueous solutions to improve the intrusion reversibility in comparison with water is of high interest for the development of new systems for mechanical energy storage. However, in some cases, the intrusion of the LiCl aqueous solutions leads to a considerable increase of the intrusion–extrusion hysteresis, thus involving the heterogeneous lyophobic systems to evolve from a spring behavior to a shock-absorber one. Nevertheless, the systems with bumper and shock-absorber behavior are promising for the applications in energy dissipation

The highest absorbed energy of 92.8 J/g was obtained for DDR-type zeosil for the first intrusion, but in this case, a combination of bumper and shock-absorber behavior is observed and the restored energy is considerably lower (31.2 J/g) [53]. Moreover, the absorbed energy value decreases strongly in 2^d^ and following cycles (60.7 J/g), even if the restored energy remains stable. For these cycles, the DDR-type zeosil presents a shock-absorber behavior. Thus, DDR-type zeosil based systems are not suitable for mechanical energy storage devices. A similar case is observed for several other systems with the highest absorbed energy values. The systems based on STF- [54] and LTA-type [46] zeosils with an absorbed energy of 40.2 and 32.6 J/g, respectively, demonstrate a combination of bumper and shock-absorber behavior in the first cycle (energy yield of 23% and 36%, respectively). In the following cycles they show a shock-absorber behavior with considerably lower absorbed energy (19.2 and 16 J/g).

The best value of stored energy among the systems with spring behavior was obtained for the “MFI-type zeosil–20 M LiCl aqueous solution” (31.3 J/g). It was tripled in comparison with water intrusion–extrusion (9.6 J/g). In order to attain systems with higher stored energy, a high energy yield, and thus a spring behavior, a study of new zeosils with a significant pore volume but low pore diameter will be of high interest for applications in the energy storage field.

## 7. Conclusions

High pressure intrusion–extrusion of LiCl aqueous solutions in hydrophobic pure silica zeolites (zeosils) were overviewed in this work. The use of lithium chloride, as well as of other salt aqueous solutions, leads to a considerable increase of intrusion pressure in comparison with water. The pressure rises with the increase of salt concentration. It was observed that the intrusion pressure of saturated LiCl aqueous solution (20 M) depends considerably on the zeosil framework. In the case of channel-type zeosils, it increases with the decrease of pore opening diameter according to the Laplace—Washburn equation, as well as in the case of water. On the contrary, no clear dependence was found, neither for pore opening size nor for the cage diameter, for the cage-type zeosils. The relative increase of intrusion pressure (P_int_ (20 M LiCl)/P_int_ (H_2_O)) is particularly strong for the cage-type zeosils with narrow pore openings (8 MR), such as LTA-, DDR-, and CHA-type zeosils (7.4, 5.95, and 5.6, respectively). Nevertheless, a strong increase (by 6.3 times) was also observed for STF-type zeosil (1D channels with side pockets, 10 MR), whereas for other zeosils with larger pore openings (10–14 MR), the relative increase is relatively low (2.0–3.3).

In several zeosils, a fully and/or partially irreversible intrusion of water is observed that corresponds to a bumper behavior or a combination of bumper and shock-absorber ones. This is related to the presence of hydrophilic silanol groups in the zeosil framework or to their formation when intruded water molecules damage the zeosil framework by breaking of siloxane bridges. The use of highly concentrated LiCl aqueous solutions instead of water leads to an increase of intrusion reversibility: the systems show a spring or a shock-absorber behavior instead of a bumper one. Depending on the zeosil structure, this effect seems to be related to two reasons. The first one is that in the case of highly concentrated solutions, most of the water molecules are included in solvation shells of lithium and chloride ions; thus, they damage the zeosil framework less, and a lower number of silanol groups is formed. The second reason is probably related to lower interactions of intruded solvated ions with silanol defects of the framework; thus, they do not remain inside the pores, when the pressure is released. For the same reason, for several zeolites which demonstrate a fully or a partially spontaneous water intrusion, high-pressure intrusion–extrusion steps appear using a concentrated LiCl aqueous solution. This increase of intrusion reversibility with LiCl aqueous solutions is of high interest for mechanical energy storage applications. However, in some cases, a considerable increase of the hysteresis between intrusion and extrusion curves is observed for highly concentrated LiCl aqueous solutions that corresponds to the transition from a spring to a bumper behavior. The highest value of absorbed energy (92.8 J/g) was obtained for the “DDR-type zeosil–20 M LiCl solution” system, but this system, as well as several other ones with the highest absorbed energy values, show a shock-absorber behavior. Thus, they are more appropriate for mechanical energy dissipation applications than for the energy storage ones. The best value of stored energy among the systems with spring behavior was obtained for the “MFI-type zeosil–20 M LiCl aqueous solution” one (31.3 J/g), where it was tripled in comparison with water. A study of new zeosils combining a significant pore volume and low pore diameter is of high interest for the applications in energy storage but also for a better understanding of the relationships between the zeosil framework and intrusion–extrusion process.

## Figures and Tables

**Table 1:** Characteristics of the frameworks of zeosils studied for water and LiCl solutions intrusion–extrusion.

Framework Type	Pore System	Ring Size (T atoms)	Average Free Diameter (Å)	Max. Diam. Includible Sphere (Å)
CDO	Multichannel (2D)	8	3.971	5.78
CHA	Cages	8	4.021	7.37
MTF	1D Channels with side pockets	8	4.113	6.25
DDR	Cages	8	4.121	7.66
LTA	Cages	8	4.157	11.05
FER	Multichannel (2D)	10 and 8	5.242	6.31
MFI	Multichannel (3D)	10	5.405	6.36
ITH	Multichannel (3D)	10 and 9	5.502	6.72
STF	1D Channels with side pockets	10	5.762	7.63
BEC	Multichannel (3D)	12	6.462	6.95
OKO	Multichannel (2D)	12 and 10	6.638	6.70
*BEA	Multichannel (3D)	12	6.709	6.68
CFI	1D Channels	14	7.976	7.47
DON	1D Channels	14	8.856	8.79

**Table 2:** Intrusion–extrusion features of zeosils under intrusion of water (0 M) and LiCl aqueous solutions. Zeosils framework types are reported specifying their porosities size R in terms of ring type (i.e., the number of T atoms constituting the ring). The following parameters are also reported: LiCl aqueous solution concentration (C.), intrusion pressure (P_int_), intruded volume (V_int_), extrusion pressure (P_ext_), extruded volume (V_ext_), absorbed (E_s_ = V_int_ × P_int_) and restored (E_r_ = V_ext_ × P_ext_) energies, energy yield (E.Y.) (Energy yield = E_r_/E_s_ ×100%), and the behavior type (SI = Spontaneus Intrusion, S = Spring, SA = Shock Absorber, B = Bumper).

			R	C (M)	P_int_ (MPa)	V_int_ (mLg^−1^)	P_ext_ (MPa)	V_ext_ (mLg^−1^)	E_int_ (Jg^−1^)	E_ext_ (Jg^−1^)	E.Y. (%)	Beh.
**1D Channels**		**CFI [50]**	14 MR	0	75	0.08	75	0.08	6.0	6.0	100	S
				10	147	0.09	143	0.09	13.2	12.9	97	S
				20	162	0.09	158	0.09	14.6	14.2	97	S
		**DON [50]**	14 MR	0	26	0.04	21	0.04	1.0	0.8	81	S
				10	81	0.06	70	0.06	4.9	4.2	86	S
				20	85	0.08	75	0.08	6.8	6.0	88	S
		**MTF [29]**	8 MR	0	125	0.008	125	0.008	1.0	1.0	100	S
				10	237	0.009	237	0.009	2.1	2.1	100	S
				15	348	0.012	348 ^I^/32 ^II^	0.007 ^I^/0.005 ^II^	4.2	2.6	62	S + SA
		**STF [54]**	10 MR	0	49 */26 **	0.055 */0.025 **	24	0.025	2.7 */0.7 **	0.6	22 */86 **	B + SA */S **
				5	120 */66 **	0.07 */0.02 **	48	0.02	8.4 */1.3 **	1	11 */72 **	B + SA */SA **
				10	180 */133 **	0.08 */0.04 **	109 */95 **	0.04	14.4 */5.3 **	4.4 */3.8 **	30 */72 **	B + SA */SA **
				20	322 */225–252 **	0.125 */0.08 **	115	0.08	40.2 */19.2 **	9.2	23 */48 **	B+ SA */SA **
**Multichannels**	**2D**	**FER [48]**	10 and 8 MR	0	150	0.056	143	0.056	8.4	8.2	97	S
				5	189	0.052	184	0.052	9.8	9.6	98	S
				10	243	0.052	231	0.052	12.6	12.0	91	S
				13	321	0.055	300	0.055	17.7	16.5	93	S
		**OKO [55]**	12 and 10 MR	0	/	/	/	/	/	/	/	SI
				20	162 */143 **	0.12 */0.105 **	131	0.105	19.4 */15.0 **	13.7	70 */98 **	B + SA */ S **
		**CDO [29]**	8 MR	0	210	0.03	180	0.03	6.3	5.4	84	S
				5	294	0.035	251	0.035	10.3	8.8	85	S
	**3D**	**ITH [52]**	10 and 9 MR	0	82	0.08	/	/	6.6	/	/	B
				5	119	0.08	/	/	9.5	/	/	B
				10	175	0.08	/	/	14	/	/	B
				20	280 */138 **	0.11 */0.06 **	117	0.06	30.8 */8.3 **	7.0	22 */84 **	B +SA */ S **
		**MFI [47]**	10 MR	0	96	0.1	95	0.1	9.6	9.5	99	S
				5	133	0.10	128	0.10	13.3	12.8	96	S
				10	193	0.10	179	0.10	19.3	17.9	93	S
				20	285	0.11	273	0.10	31.3	27.3	87	S
		*BEA [44]	12 MR	0	53	0.14	/	/	8.3	/	/	B
				10	95	0.12	/	/	11.4	/	/	B
				15	111	0.16	102	0.16	17.8	16.3	91	S
				20	115	0.16	103	0.16	18.4	16.5	90	S
		**BEC [51]**	12 MR	0	41	0.08	/	/	3.3	/	/	B
				20	124 */119 **	0.11	82	0.11	13.6 */13.1 **	9.02	66 */69 **	SA
**Cages**		**DDR [53]**	8 MR	0	60	0.112	51	0.112	6.7	5.7	85	S
				10	193 */166 **	0.08 */0.07 **	166	0.07	15.4 */11.6 **	11.6	75 */100 **	B + SA */S **
				20	357 */253 **	0.26 */0.24 **	130	0.24	92.8 */60.7 **	31	33 */51 **	B + SA */SA **
		**CHA [49]**	8 MR	0	29 */22 **	0.15 */0.13 **	22 */20 **	0.13	4.4 */2.9 **	2.9 */2.6 **	65 */90 **	B + SA */S **
				5	66 */63 **	0.15	54	0.15	9.9 */9.4 **	8.1	82 */86 **	S
				10	90 */86 **	0.15	79	0.15	13.5 */12.9 **	11.8 */11.8 **	88 */92 **	S
				20	162 */153 **	0.15	137	0.15	24.3 */22.9 **	20.5	85 */89 **	S
		**LTA [46]**	8 MR	0	20	0.17	/	/	3.4	/	/	B
				10	53 */46 **	0.20 */0.12 **	39	0.12	10.6 */5.5 **	4.7	42 */85 **	B + SA */S **
				20	148 */133 **	0.22 */0.12 **	98	0.12	32.6 */16.0 **	11.8	36 */74 **	B + SA */SA **

The results obtained in the first and the following cycles are indicated by * and ** respectively. The index I and II correspond to the 1st and 2nd extrusion steps in MTF-type zeosil. The behaviors indicated in the table can be different from the ones of corresponding references, since the attribution of spring and shock-absorber behavior has been changed (S if E.Y. > 80%, SA if E.Y. < 80%).
